# Grip force when reaching with target uncertainty provides evidence for motor optimization over averaging

**DOI:** 10.1038/s41598-017-10996-6

**Published:** 2017-09-15

**Authors:** Joseph Y. Nashed, Jonathan S. Diamond, Jason P. Gallivan, Daniel M. Wolpert, J. Randall Flanagan

**Affiliations:** 1 Centre for Neuroscience Studies, Queen’s University, Kingston, Ontario, Canada; 2 Department of Psychology, Queen’s University, Kingston, Ontario, Canada; 3 Department of Psychology, Queen’s University, Kingston, Ontario, Canada; 4 Department of Engineering, University of Cambridge, Cambridge, UK

## Abstract

When presented with competing potential reach targets and required to launch a movement before knowing which one will be cued as the target, people initially reach in the average target direction. Although this spatial averaging could arise from executing a weighted average of motor plans for the potential targets, it could also arise from planning a single, optimal movement. To test between these alternatives we used a task in which participants were required to reach to either a single target or towards two potential targets while grasping an object. A robotic device applied a lateral elastic load to the object requiring large grip forces for reaches to targets either side of midline and a minimal grip force for midline movements. As expected, in trials with two targets located either side of midline, participants initially reached straight ahead. Critically, on these trials the initial grip force was minimal, appropriate for the midline movement, and not the average of the large grip forces required for movements to the individual targets. These results indicate that under conditions of target uncertainty, people do not execute an average of planned actions but rather a single movement that optimizes motor costs.

## Introduction

Studies of goal-directed reaching have typically focused on the planning of movements towards a single target. However, in many real world action tasks, we need to decide which, of several potential reach targets available in the environment, to act upon. According to traditional serial models, a final target is first selected and only then is the sensorimotor system engaged to plan a movement to the selected target^1–3^. However, based on mounting neural^4–6^, and behavioural^7–10^ evidence, this serial view of action planning has been challenged. Specifically, it has been proposed that, under conditions of target uncertainty, the brain prepares multiple actions, in parallel, for competing potential targets before selecting one of them to execute^4,11^. This proposition resonates with Gibson’s^12^ influential idea that a necessary aspect of object perception is specifying the potential actions afforded by objects in our environment.

Much of the behavioural evidence for the multiple plans hypothesis is based on ‘go-before-you-know’ tasks in which participants are required to launch a movement towards two or more potential targets, one of which is selected (cued) as the target after movement onset. These studies have shown that the initial direction of the launched movement closely corresponds to a spatial average of the movements generated for the potential targets when presented individually^7,10,13–17^. Although such motor averaging has been taken as evidence that the motor system forms, and then averages, multiple motor plans for the potential targets^14,16^, it is also consistent with the idea that the motor system plans and executes a single movement that minimizes the cost of corrective movements required after the target is cued^18–20^.

To investigate this issue, we designed a go-before-you-know task in which participants generated hand movements while grasping an object instrumented with sensors to measure grip and load forces. In one-target trials, the target was located in one of three directions, either straight-ahead (0°) or left or right of straight-ahead (±30°). In two-target trials, in which the final target was uncertain at movement onset, both the lateral targets were presented and participants were required to launch a movement before knowing which potential target would be cued as the target. A lightweight robotic device, attached to the object, applied a lateral elastic load toward the midline proportional to the distance of the object from the midline. Thus, whereas substantial loads were experienced in one-target trials with the lateral targets, minimal loads were experienced for the midline target, and when initially reaching straight ahead in two-target trials. Based on previous work, we expected that participants would readily learn the loads associated with these one-target movements, and would express this learning by precisely modulating grip force in synchrony with, and thus in anticipation of, the load force^21–24^. We reasoned that if initial movements in two-target trials directly represent the execution of two motor plans associated with each lateral target, then grip force should be elevated when initially reaching straight ahead, even though the load would be minimal. However, if participants generate a single optimal movement—or an approximation of an optimal movement—in two-target trials, then the grip force should be predictively scaled for the minimal, expected load.

## Methods

### Participants

Five men and 4 women between the ages of 20 and 28 (M_age_ = 23.75, SD_age_ = 2.43) years of age participated in the present study after providing written, informed consent. All participants self-reported having normal or corrected-to-normal vision, being right handed, and being free of sensorimotor dysfunction. All participants completed the same conditions and same trial order. All but one participant exhibited robust spatial averaging behaviour whereby, when required to launch a movement in response to the presentation of two potential targets, the initial movement direction was midway between the two target directions. However, one participant tended to aim their initial movement towards one of the two potential targets. Because our research question focused on trials with spatial averaging, this one participant was excluded from further analysis. The experimental protocol was approved by the General Research Ethics Board at Queen’s University in compliance with the Canadian Tri-Council Policy on Ethical Conduct for Research Involving humans. Each experimental session lasted approximately one hour and participants were compensated $10 per hour of participation.

### Apparatus and Stimuli

**Figure 1:**
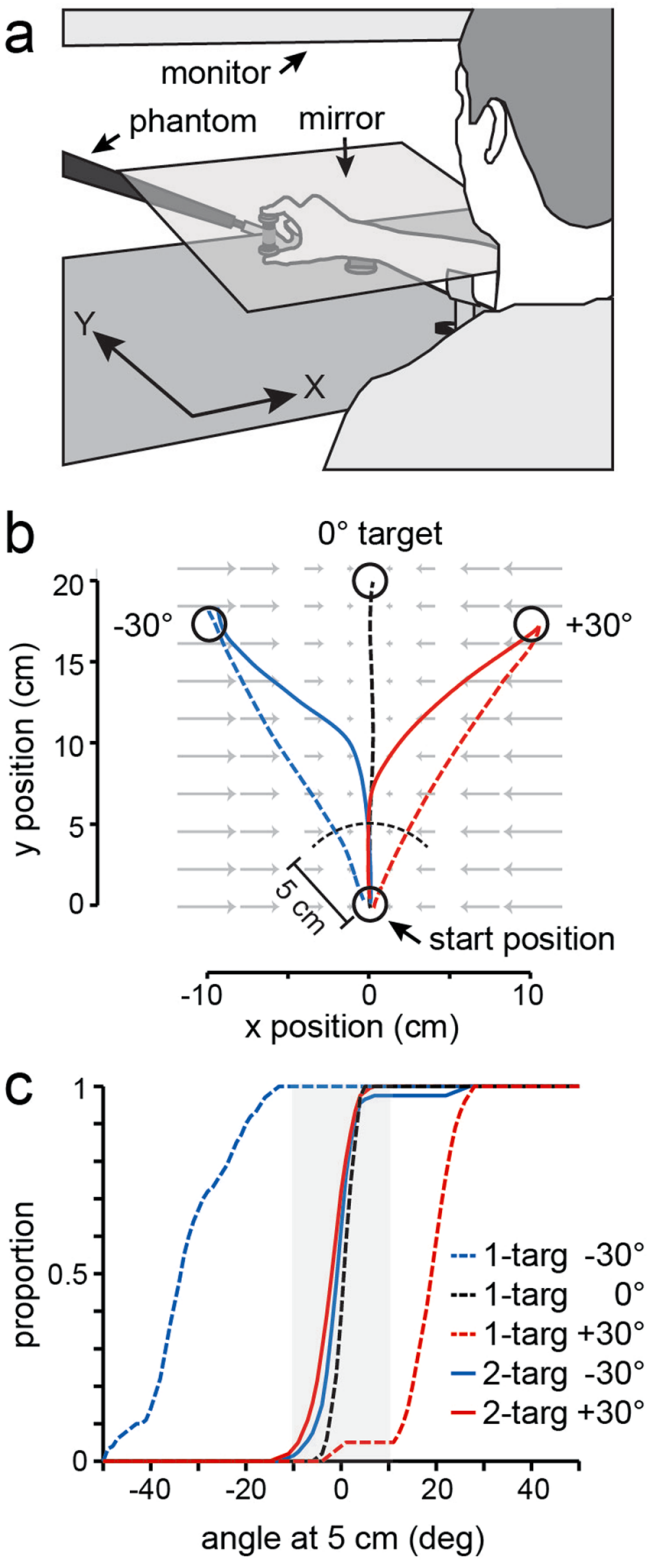
(**a**) Participants moved an object, held using a precision grip, towards targets presented in the horizontal plane. The object was attached to a robotic device that generated position-dependent lateral forces. (**b**) Object paths for 3 one-target trials and 2 two-target (±30°) trials in which either the −30 or the +30° potential target was cued as the target after movement onset. Grey arrows shows elastic forces. (**c**) Cumulative frequencies showing the angle of the object, relative to the start position, at 25% of the distance to the target arc (5 cm arc shown in panel b) in one-target and two-target trials. Data from all trials from all participants included. The line types in the legend apply to both **b** and **c**.

Participants performed reaching movements towards targets in the horizontal plane while grasping an object with a vertically oriented grip (with the index finger and thumb contacting flat circular (2.5 cm in diameter) surfaces located on the top and bottom of the object, respectively, and positioned 6.4 cm apart (see Fig. 1a). Each object surface was covered in sandpaper and mounted on a six-axis force-torque transducer (Nano force/torque, ATI Industrial Automation, Apex, NC), which measured forces and torques in three dimensions. The center of the object was attached to a lightweight robotic device (Phantom 3.0 haptic interface, Sensable, Wilmington, MA) through a two-joint linkage, and the grip surfaces freely rotated about the long axis of the object. This construction allowed full three-dimensional rotation of the object. Encoders in the robotic device recorded the position of the object in three dimensions. The experimenter monitored the orientation of the object throughout testing to ensure that the object, and grip axis, remained vertical. Two air-sleds fitted with cuffs were used to support the arm slightly below the elbow and at the wrist, allowing near-frictionless motion of the arm in the horizontal plane. The targets, start position, and a cursor representing the position of the grasped object, were displayed on a 30-inch monitor, located above the plane of movement, and viewed through a mirror positioned halfway between the plane of movement and the monitor, and which occluded vision of the hand and arm. Thus, these images appeared in the movement plane.

In the main experimental condition, the robotic device was used to simulate an elastic load acting on the object whereby the force applied by the object to the hand increased in proportion to the lateral (x) displacement of the object from midline, and was directed towards the midline (see grey arrows in Fig. 1b). Specifically, the lateral force was defined as *F*_*x*_ = −*k* x where the stiffness, *k*, was equal to 0.73 N/cm with the midline at x = 0. Note that with this load, or force field, little or no force was applied to the object during straight-ahead, midline movements. In the control condition, no load was applied by the robot to the object.

### Procedure

To begin each trial, the participant positioned the object cursor (0.5 cm in diameter) at the circular start position (1 cm in diameter) located approximately 30 cm in front of the participant at midline. Once the cursor was held at the start target for 200 ms, either one target or two potential targets were displayed. All targets were located 20 cm from the start position and were initially displayed as open circles (1 cm in diameter; see Fig. 1b). In one-target trials, the target could be presented at one of three possible locations: −30°, 0°, or +30°. In all two-target trials, the potential targets were presented at −30° and 30°. The presentation of the target(s), concurrent with an auditory beep cue, provided the ‘go’ signal prompting the participant to initiate a reaching movement towards the target(s). Critically, in two-target trials, the actual target was only cued (filled in) when movement onset was detected, that is when the center of the grasped object left the start target. For consistency, in one-target trials, the target was also filled in at movement onset.

Each participant first completed a task training phase in which they practised the task without a load applied. This phase included 60 one-target trials (20 per target position) and 80 two-target trials (40 per cued target), with all trials randomly intermixed. This was followed by a load familiarization block of 30 one target trials (10 per target position) with the elastic load applied. Participants then performed the experimental condition which was identical to the training phase (albeit in a different random order) except that the elastic force field was implemented.

### Data analysis

The position of the object and forces applied by the hand to the force sensors were recorded at 1000 Hz. These signals were digitally smoothed using a fourth-order dual-pass Butterworth filter with a cutoff frequency of 14 Hz. The grip force applied to the object was taken as the normal force applied to the force sensor contacted by the index finger. The load force acting on the object was computed as the resultant of the two tangential forces applied to the same force sensor, multiplied by two. This load force measured the elastic (i.e., position-dependent) force applied by the robot to the object as well as a small inertial (i.e., acceleration dependent) component associated with the mass of object. We will use the term ‘elastic force’ to refer to the commanded force sent to the robotic device, and the term ‘load force’ to refer to the tangential force applied to the sensor.

To examine the direction of the initial hand movement in two-target trials, we determined the angle (relative to midline or 0°) of the vector from the start position to the position of the object when it reached 25% of the distance (i.e., 5 cm) to the arc upon which the targets were located. As will be shown below, this ensured that we measured the direction before corrective movements could be made in response to the cueing of the target at movement onset. To assess the grip force and load force of this initial movement, we also determined these forces at the time at which the object reached 25% of the distance to the target arc.

Planned comparisons were used to compare kinematic and force variables across the five target types: the −30, 0 and +30° targets for one-target trials and the −30 and +30° targets for two-target trials. A p-value of ≤0.05 was considered to be significant.

## Results

Single movement paths from a representative participant are shown in Fig. 1b. The dashed lines show paths in the three types of one-target trials (i.e., with the −30°, 0°, and +30° targets) and the solid lines show paths for the the two types of two-target trials (with either the −30° or +30° target being cued at movement onset). As illustrated in this figure, roughly straight-line paths were produced in the one-target trials, and the initial path in two-target trials tended to be a spatial average of the −30° or +30° movement paths (i.e., initially directed straight ahead or ~0°) before correcting towards the cued target well after the handle had travelled 5 cm from the start position.

Figure 1C shows cumulative distributions, combining all trials from all participants, of the initial movement direction (i.e., the angle of the handle, relative to the start position, when the handle had travelled 5 cm) for the three types of one-target trials and the two types of two-target trials. For all participants, the direction of initial movement in two-target trials was within ±10° of the straight-ahead direction (0°) in the vast majority of trials (see grey area). Because we were interested in examining grip force in the approximately straight-ahead movements in two-target trials (i.e., movements exhibiting spatial averaging), we excluded all two-target trials in which the initial movement direction did not fall within the ±10° window. This resulted in the exclusion of <5% of these trials.

**Figure 2:**
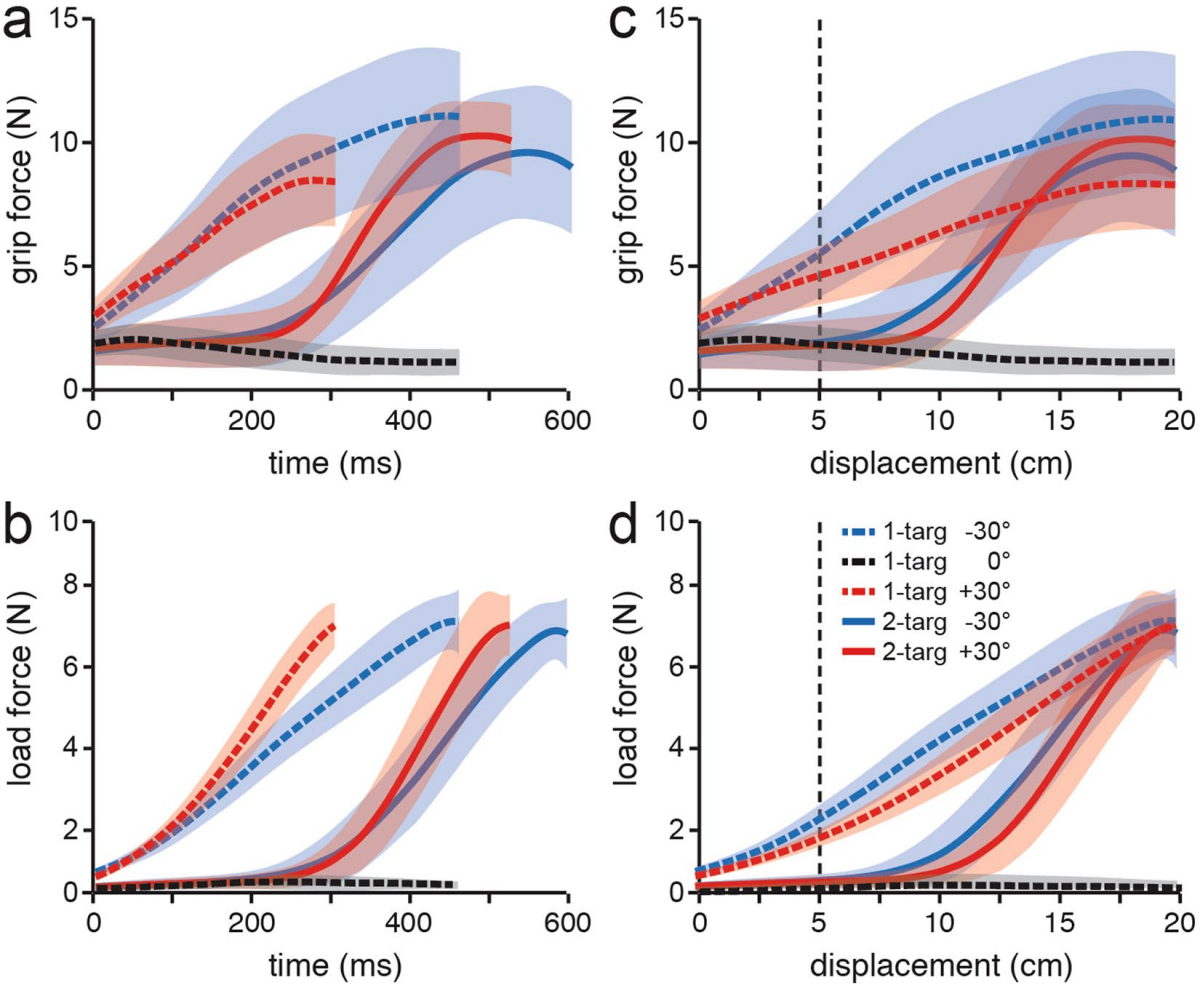
Mean grip (**a**) and load forces (**b**) as a function of time from movement onset, from a single participant, for the 3 types of one-target trials and the 2 types of two-target trials. For each trial type, the average was computed from trials normalized, in time, to the median movement duration of that trial type. Corresponding mean grip and load forces as a function of displacement (**c** and **d**). The vertical dashed lines represent the point at which the handle moved 25% of the distance to the target arc (5 cm). The shaded regions represent ±1 s.d. The line types in the legend apply to all panels.

Figure 2 shows average grip and load forces both as a function of time relative to movement onset (Fig. 2a,b) and movement displacement (Fig. 2c,d), for the same representative participant shown in Fig. 1b. Separate averages are shown for each of the 5 trial types. For the time-varying plots, the trials were normalized to the median duration, from movement onset until the handle reached the target arc, for all of the trials of that trial type. As exemplified by this participant, movement duration at the group-level was greater for two-target trials than one-target trials (t_7_ = 6.643; p < 0.001), which is expected given the longer hand paths observed in two-target trials. We also found that for both one-target (t_7_ = 4.580; p < 0.01) and two-target (t_7_ = 4.559; p < 0.01) trials, movement duration was shorter for movements terminating at the +30° target in comparison to those terminating at the −30° target. However, the duration of the initial 25% of the movement in two-target trials was shorter than in one-target trials with the 0° target, where similar load forces were observed (F_1, 7_ = 147; p < 0.001). (Note that these significant effects, as well as all significant effects reported below, remained highly significant with a Bonferroni correction for multiple comparisons).

Because hand paths in one-target trials were fairly straight, load force increased monotonically as a function of displacement in one-target trials directed to the ±30° targets, but remained very low in one-target trials directed to the 0° target (Fig. 2). As expected, the pattern of grip force in these one-target trials was similar to the load force, increasing monotonically as a function of displacement for movements to the ±30° targets and remaining low for movements to the 0° target. Critically, in two-target trials, the grip force over the initial 25% of the movement (see vertical dashed lines in Fig. 2c,d) remained low and only began to increase sharply when the load force began to increase during the subsequent corrective movement. Thus, as in the one-target trials, grip force in two-target trials in this participant was modulated in synchrony with load force, and the grip force observed over the initial component of the movement was clearly far lower than the average of the initial grip forces in the one-target trials to the ±30° targets.

**Figure 3:**
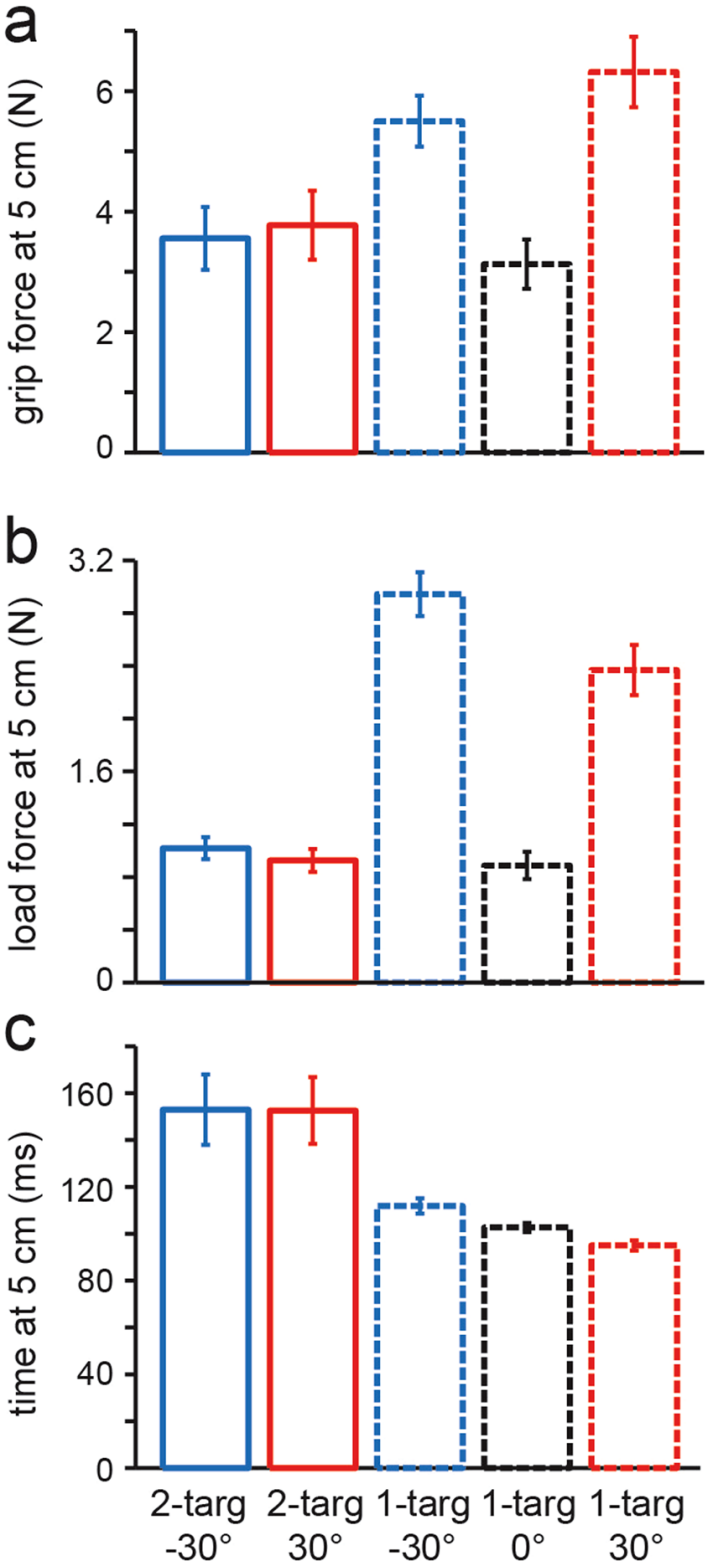
Average grip force (**a**), load force (**b**) and time relative to the start of the movement (**c**), based on participant means, at the point at which the object reached 25% of the distance to the distance to the target arc (5 cm) in the 3 types of one-target trials and the 2 types of two-target trials (see line types in legend). The vertical lines represent ±1 s.e.

Figure 3 shows, for each of the five trial types, the average grip force, load force and movement time, based on participant means, when the handle reached 25% of the distance (5 cm) to the target arc. For both grip force and load force, we carried out a 2 × 2 repeated measures ANOVA with number of targets (one or two) and cued target (±30°) as independent variables. In addition, we carried out a contrast comparing the two types of two-target trials to the 0° one-target trials. As expected, for initial load force the 2 × 2 ANOVA revealed a highly significant effect of number of targets (F_1, 7_ = 266; p < 0.001) but not an effect of cued target (F_1, 7_ = 3.26; p = 0.114) and no interaction (F_1, 7_ = 2.42; p = 0.164). In addition, the contrast failed to reveal a reliable difference between the two-target trials and the 0° one-target trials (F_1, 7_ = 0.68; p = 0.415). The latter result is consistent with the observation that the initial movement in two-target trials was consistently aimed in the straight-ahead (0°) direction.

For initial grip force, like load force, the 2 × 2 ANOVA revealed an effect of number of targets (F_1, 7_ = 21.2; p = 0.002), but no effect of cued target (F_1, 7_ = 3.98; p = 0.086) and no interaction (F_1, 7_ = 2.50; p = 0.158). Like load force, the contrast failed to reveal a significant difference between the two-target trials and the 0° one-target trials (F_1, 7_ = 1.16; p = 0.317). These results support the observation that the initial grip force (i.e., at 25% of target distance) in two-target trials was less than the average of grip forces in the corresponding one-target trials involving the +30 and −30° targets, and, in fact, was no different than the grip force in one-target trials involving the 0° target.

## Discussion

Here we tested whether individuals, when required to move a grasped object towards two simultaneously presented potential targets prior to knowing which will be selected as the target, execute a weighted average of two planned actions, formed for each target, or produce an initial movement that instead optimizes motor costs. As expected, we found that the initial movement was spatially directed midway between the two targets. However, the initial grip force predictively scaled with the minimal load forces associated with this intermediate movement direction, and not an average of the grip forces required for the two movements associated with the potential targets. This result supports the idea that, when presented with multiple competing action options, participants generate a single movement, in an optimal fashion, prior to implementing a corrective action towards the selected target.

Previous work examining movement planning in cases of target uncertainty has interpreted reach averaging behaviour, in which initial reaches are directed midway between the distribution of the multiple presented targets^13–16^, as evidence that the motor system forms multiple potential motor plans, and executes a weighted average of these plans^16^. However, such behaviour is also broadly consistent with planning a single movement that tends to minimize the motor costs of corrective actions required once a target is cued after movement onset^15,25^. In a previous study using a go-before-you-know task, we attempted to dissociate the average reach direction from the average target direction^7^. Two potential targets were located either side of midline and an obstacle was placed between the start position and the right target such that the initial hand direction when reaching directly to the right target (when presented alone) was straight ahead. The initial movement in uncertain trials was in the average reach direction—to the left of midline and biased away from the obstacle—and not the average target direction, even though the latter was optimal in terms of movement corrections. Although this result appears to favour motor averaging over planning a single optimal movement, given the variability of initial movement directions observed in uncertain trials, it is possible that participants planned a single movement that balanced optimizing the cost of corrections and avoiding the obstacle^10^.

The idea that motor plans are averaged has been challenged by recent work demonstrating that averaging behaviour is less likely to be observed when the benefit of reaching in an intermediate direction is reduced either by increasing the angular separation between targets^19^ or by increasing required movement speed^20^ (see also^13^). These findings have been taken as evidence that the brain, rather than executing an average of two competing movements, instead specifies a single movement that optimizes task success^19,20^. A recent study^10^ showing that potential targets are rapidly and automatically transformed into motor representations suggests that such optimal planning is based, at least in part, on movement-related parameters (e.g., final hand position or hand direction) associated with the targets rather than visual parameters (see also^25^).

As noted above, the minimal grip force observed during the initial, straight ahead movement in two-target trials suggests that participants did not execute an average of two straight line movements to these targets. However, if participants planned curved, initially straight movements for each target and then averaged these two curved movements, the initial grip force would be expected to be minimal. Although we cannot rule out this scenario, we feel it is unlikely because it would involve planning different (i.e., straight versus curved) movements for a given lateral target depending on whether that target is presented alone or simultaneously with the other lateral target. However, there is evidence that alternative corrective actions, which in our task are launched once the target is cued after movement onset in two-target trials, may be prepared, or ‘precomputed’, in advance^26^. In the context of object manipulation tasks, there is strong evidence that the sensorimotor system prepares alternative, task-protective corrective actions that can be triggered by new state information^27^.

Recent behavioural studies have provided evidence that in situations in which a movement is executed *after* one of two potential target is cued as the target, people prepare competing motor plans prior to target selection^8,28^. This hypothesis is supported by neurophysiological studies, using ‘go-after-you-know’ tasks, showing that competing potential targets elicit separate neural representations in areas involved in action—including dorsal premotor cortex and the parietal reach region— prior to one of the targets being selected^4–6^. Thus, although the current findings suggest that people do not execute a weighted average of competing motor plans when required to rapidly launch a movement before knowing which potential target will be selected as the target, it is entirely possible that, under different circumstances, the brain does specify competing actions. Specifically, in go-after-you-know tasks, in which there is sufficient time between the presentation of potential targets and movement execution, competing actions may be prepared in advance of action selection. This may allow potential actions to be launched more quickly and reduce the cognitive load associated with maintaining multiple potential targets in working memory^8^. Likewise, in free choice scenarios, it may also allow motor-related costs to be factored into decision processes related to target and action selection^29–31^.

### Data availability statement

The authors are happy to make the data used in this report upon request.

## References

[1] Sternberg S (1969). Memory-scanning: mental processes revealed by reaction-time experiments. Am. Sci..

[2] Miller, G. A., Galanter, E. & Pribram, K. H. *Plans and the structure of behavior*. (Henry Holt, 1960).

[3] McClelland JL (1979). On the time relations of mental processes: An examination of systems of processes in cascade. Psychol. Rev..

[4] Cisek P, Kalaska JF (2005). Neural correlates of reaching decisions in dorsal premotor cortex: specification of multiple direction choices and final selection of action. Neuron.

[5] Cui H, Andersen RA (2011). Different representations of potential and selected motor plans by distinct parietal areas. J Neurosci.

[6] Klaes C, Westendorff S, Chakrabarti S, Gail A (2011). Choosing goals, not rules: deciding among rule-based action plans. Neuron.

[7] Stewart BM, Gallivan JP, Baugh LA, Flanagan JR (2014). Motor, not visual, encoding of potential reach targets. Curr. Biol..

[8] Gallivan JP, Barton KS, Chapman CS, Wolpert DM, Randall Flanagan J (2015). Action plan co-optimization reveals the parallel encoding of competing reach movements. Nat. Commun..

[9] Gallivan JP, Logan L, Wolpert DM, Flanagan JR (2016). Parallel specification of competing sensorimotor control policies for alternative action options. Nat. Neurosci..

[10] Gallivan JP, Stewart BM, Baugh LA, Wolpert DM, Flanagan JR (2017). Rapid Automatic Motor Encoding of Competing Reach Options. Cell Rep..

[11] Cisek P, Kalaska JF (2010). Neural mechanisms for interacting with a world full of action choices. Annu. Rev. Neurosci..

[12] Gibson, J. J. The Ecological Approach to Visual Perception. (1979).

[13] Ghez C (1997). Discrete and continuous planning of hand movements and isometric force trajectories. Exp. Brain Res..

[14] Gallivan JP (2011). One to four, and nothing more: nonconscious parallel individuation of objects during action planning. Psychol. Sci..

[15] Stewart BM, Baugh LA, Gallivan JP, Flanagan JR (2013). Simultaneous encoding of the direction and orientation of potential targets during reach planning: evidence of multiple competing reach plans. J. Neurophysiol..

[16] Chapman CS (2010). Reaching for the unknown: multiple target encoding and real-time decision-making in a rapid reach task. Cognition.

[17] Gallivan JP, Chapman CS (2014). Three-dimensional reach trajectories as a probe of real-time decision-making between multiple competing targets. Front. Neurosci..

[18] Hudson TE, Maloney LT, Landy MS (2007). Movement planning with probabilistic target information. J. Neurophysiol..

[19] Haith AM, Huberdeau DM, Krakauer JW (2015). Hedging your bets: intermediate movements as optimal behavior in the context of an incomplete decision. PLoS Comput Biol.

[20] Wong AL, Haith AM (2017). Motor planning flexibly optimizes performance under uncertainty about task goals. Nat. Commun..

[21] Flanagan JR, Wing AM (1997). The role of internal models in motion planning and control: evidence from grip force adjustments during movements of hand-held loads. J. Neurosci..

[22] Flanagan JR, Vetter P, Johansson RS, Wolpert DM (2003). Prediction Precedes Control in Motor Learning. Curr. Biol..

[23] Diamond JS, Nashed JY, Johansson RS, Wolpert DM, Flanagan JR (2015). Rapid Visuomotor Corrective Responses during Transport of Hand-Held Objects Incorporate Novel Object Dynamics. J. Neurosci..

[24] Danion F, Diamond JS, Flanagan JR (2013). Separate contributions of kinematic and kinetic errors to trajectory and grip force adaptation when transporting novel hand-held loads. J. Neurosci..

[25] Christopoulos V, Schrater PR (2015). Dynamic Integration of Value Information into a Common Probability Currency as a Theory for Flexible Decision Making. PLoS Comput. Biol..

[26] Nashed JY, Crevecoeur F, Scott SH (2014). Rapid online selection between multiple motor plans. J. Neurosci..

[27] Johansson RS, Flanagan JR (2009). Coding and use of tactile signals from the fingertips in object manipulation tasks. Nat. Rev. Neurosci..

[28] Gallivan JP, Bowman NAR, Chapman CS, Wolpert DM, Flanagan JR (2016). The sequential encoding of competing action goals involves dynamic restructuring of motor plans in working memory. J. Neurophysiol..

[29] Cos I, Bélanger N, Cisek P (2011). The influence of predicted arm biomechanics on decision making. J. Neurophysiol..

[30] Cos I, Duque J, Cisek P (2014). Rapid prediction of biomechanical costs during action decisions. J. Neurophysiol..

[31] Morel P, Ulbrich P, Gail A (2017). What makes a reach movement effortful? Physical effort discounting supports common minimization principles in decision making and motor control. PLoS Biol..

